# Electrospray Ion Mobility Spectrometer Based on Flexible Printed-Circuit Board Electrodes with Improved Resolving Power

**DOI:** 10.1021/acs.analchem.3c01898

**Published:** 2023-07-05

**Authors:** Marc-Aurèle Boillat, Julian M. Rakus, Peter C. Hauser

**Affiliations:** Department of Chemistry, University of Basel, Klingelbergstrasse 80, 4056 Basel, Switzerland

## Abstract

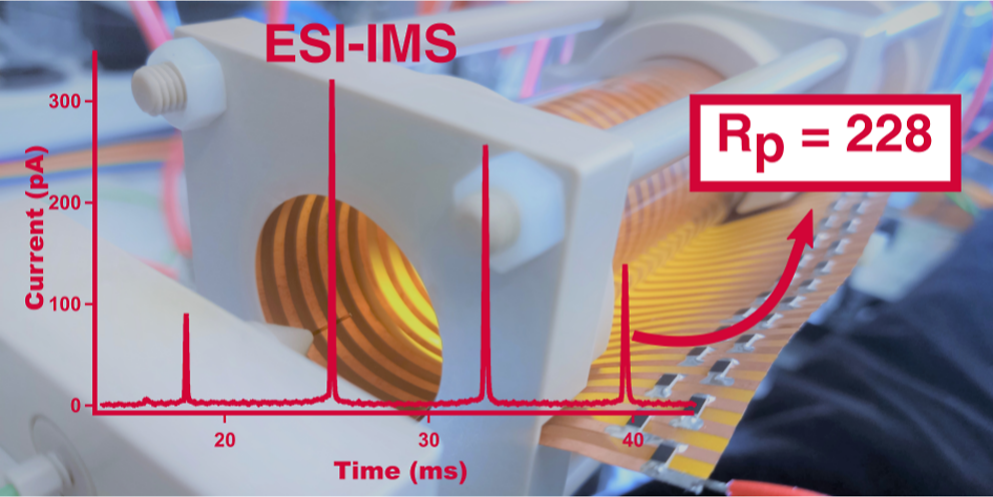

An easily built drift tube instrument with ring electrodes made of rolled-up flexible printed circuit boards is reported. Its resolving power was maximized by careful attention to the drift tube geometry and the response time of the detector amplifier and by employing a high separation field strength. The separation of singly charged aliphatic quaternary ammonium ions introduced by electrospray was performed, and the measured resolving power was between 86 and 97% of the theoretical limit for three different drift tube lengths investigated. For the longest drift length of 30 cm, a resolving power of up to 228 was obtained. Three benzalkonium chlorides were also separated with resolving powers of over 210. The tristate injection scheme can also be used, with only a small loss of the separation performance compared to the two-state injection.

## Introduction

Ion mobility spectrometry (IMS) is a gas-phase analytical technique that separates ions based on their mobility by driving them through a buffer gas using an electric field. The different drift velocities of the ions are related to their charge and size and are responsible for their separation in the gas phase in a drift tube.^1^ Owing to its short analysis time and low limit of detection, IMS has been extensively used for the detection of explosives and chemical weapons at atmospheric pressure.^2^ But its field of applications has also been expanded to pharmaceutical, food, and environmental analyses.^3^ Various ion sources have been used with IMS, including radioactive ionization,^2^ microplasmas,^4−6^ and electrospray ionization (ESI),^7,8^ among others.

One focus of research in IMS has been the improvement in the separation performance. This is an important challenge as, indeed, the most popular implementation of this technique, drift-tube IMS (DTIMS), often shows limited separation capabilities. DTIMS is characterized by pulsed injection of ion packets into a drift tube with a uniform field along its axis, which allows the direct determination of ion mobility.^9^ The separation performance of atmospheric pressure DTIMS can be optimized mainly by using short injection times (at the cost of sensitivity) and using high field strengths in short drift tubes.^10^ Alternatively, the drift time and/or drift distance are increased by making use of alternative modes of IMS. This comprises trapped IMS,^11^ ion cyclotron IMS,^12^ and traveling wave IMS.^13^ However, these methods require a radial confinement of the ions in order to suppress losses on the wall. This can be achieved by application of a radiofrequency alternating current field but necessitates working at reduced pressures. Both of these measures significantly increase the complexity of the instrumentation.

The separation efficiency is usually expressed in terms of resolving power, *R*_p_, which is defined as the peak arrival time, *t*_d_, divided by the peak full width at half maximum (FWHM), *w*_0.5_
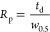
1

Traditionally, IMS instruments with resolving powers above 80 and above 200 have been classified, respectively, as “high-resolution” and “ultrahigh-resolution”.^14^ Singly charged ions (*z* = 1) are known to be more difficult to separate than multiply charged ions, so that for the latter higher resolving powers are generally obtained.^10,15^ Among the atmospheric pressure DTIMS instruments reported in the literature to date, only very few may be considered to fall under the definition of ultrahigh resolution. Using an ESI source and MS detection, best case resolving powers of 216 (*z* = 11) and 240 (*z* = 4) were reported by Srebalus *et al.*(16) and Wu *et al.*,^7^ respectively, for large biomolecules with multiple charges. With a pulsed laser source, C_60_^+^ fullerene clusters were detected with a resolving power of 172 in a 63 cm long drift tube coupled to MS and operated at 500 Torr.^17^ A resolving power of 250 for singly charged ions was reported by Zimmermann’s group using a radioactive ion source^15^ and UV ionization.^18^ This was achieved mainly by using a purpose-built instrument with a drift tube of 15 cm length, which is slightly longer than the typical 10 cm, and the use of a very short injection time of 5 μs. Very recently, Zimmermann’s group also demonstrated a resolving power of 155 for an ESI–IMS.^19^ This was achieved in a drift tube of 75 mm length by using a short injection time of 5 μs and an elevated drift tube pressure of 1802 hPa.

A further line of research has been the development of DTIMS instruments whose construction is simple and inexpensive. As the implementation of DTIMS is much more straightforward than that of other analytical techniques, such as mass spectrometry or liquid chromatography, in-house building of instruments in the spirit of open-source hardware is possible. Drift tubes for IMS have traditionally been made from alternately stacked rings of electrodes and insulators, which have to be machined individually in a mechanical workshop. Various simplified designs based on rigid printed circuit boards (PCBs) have been reported in recent years.^20−22^ These can be produced with standard techniques as employed in the electronics industry and ordered readily from a multitude of PCB manufacturers. Smith *et al.*(23) and Chantipmanee and Hauser^24^ described DTIMS instruments whose drift electrodes were tracks on the flexible PCB material, which was rolled into cylindrical drift tubes of 12 and 10 cm length, respectively. These designs reached resolving powers of 82 and 85. Recently, Clowers’ group^25^ carried out a comparison of do-it-yourself instruments based on stacked rigid PCBs and on flexible PCBs. Similar resolving powers were reported for a range of quaternary ammonium ions introduced with ESI with a slightly lower performance for the drift tube based on the flexible PCB.

Herein, the improvement of the resolving power of an in-house-built ESI–DTIMS, based on rolled-up flexible PCBs as electrodes, and ESI is described. This was achieved by careful re-examination and optimization of different aspects that might impact the resolving power. The design of the instrument follows the open-source hardware notion.

The theoretically achievable resolving power, *R*_p_, can calculated by expanding eq 1 to derive the drift time from the mobility, *K*, the length of the drift tube, *L*, and the applied voltage, *V*_D_. The expected value for *w*_0.5_ can be obtained from the geometrical mean of the injected width, *w*_inj_, and a term for diffusional broadening, as described by Revercomb and Mason.^26^ A further term accounting for any broadening caused by the response time of the detector amplifier can be added, as suggested by Kirk *et al.*(27)
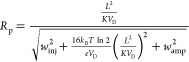
2Here, *T* is the temperature, *k*_B_ is the Boltzmann constant, and *e* is the elementary charge.

Best resolving powers can, therefore, be achieved by optimizing the variable experimental parameters of injection time, length of the drift tube, applied voltage, and bandwidth of the detector. However, the equation does not account for possible additional causes of band broadening, such as inhomogeneity of the field in the drift tube and imprecise alignment (parallelism) of grids and detector.^28,29^ A further important point to consider is the signal-to-noise ratio of the measurement, which will depend on the detector sensitivity and the amount of analyte available for detection. The latter is determined by the density of the ion cloud available for injection and the injection time, as well as losses during the drift to the detector through radial diffusion to the wall and possible other mechanisms.

## Experimental Section

### Chemicals

The following compounds were obtained as bromide salts from Sigma-Aldrich (Buchs, Switzerland): tetraethylammonium (T2), tetrabutylammonium (T4), tetrahexylammonium (T6), tetraoctylammonium (T8), tetradecylammonium (T10), and tetradocecylammonium (T12). Benzyldimethyldodecylammonium (BAC-C12), benzyldimethyltetradecylammonium (BAC-C14), and benzyldimethylhexadecylammonium (BAC-C16) chloride salts were also purchased from Sigma-Aldrich. Standards with a concentration of 5 μM were prepared in 50% v/v methanol (HPLC grade, HiPerSolv, VWR Chemicals, Schlieren, Switzerland) and water with a resistivity of 18.2 MΩ·cm (Milli-Q, Merck Millipore, Schaffhausen, Switzerland) from 1 mM stock solutions.

### Instrumentation

**Figure 1:**
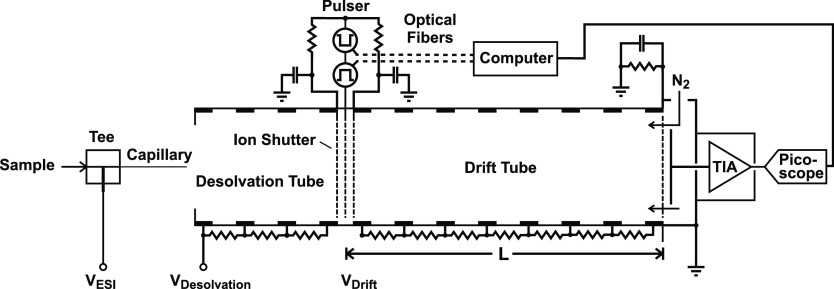
Schematic diagram of the IMS setup.

The IMS instrument was constructed in-house based on the flexible PCB design previously reported by our group.^24^ The main modifications consisted in the adaptation of the components to the higher voltages applied and the lengthening of the drift tube. In addition, the experimental setup was placed on a custom-made poly(methyl methacrylate) (PMMA) breadboard. Cables specified for 30 and 40 kV (HTV-30S-22-2 and HSW-4022-2, Hivolt.de, Hamburg, Germany) were employed for the high voltage connections. A schematic diagram of the spectrometer is shown in Figure 1. The desolvation and drift tubes were etched from flexible copper-clad polyimide (Goodfellow, Huntingdon, UK) as previously described.^24^ Their respective lengths were 10.0 and 30.4 cm. The 1.6 mm wide copper tracks forming the electrodes were separated by 2.0 mm gaps and connected through surface-mount 1 MΩ resistors (Stackpole Electronics, Digi-Key Electronics, Thief River Falls, MN). These flexible PCBs were rolled up into slotted PMMA tubes to adopt a cylindrical shape. A rigid frame composed of polyether ether ketone (PEEK) flanges held by four PEEK rods was used to support the tubes. This frame was designed to accommodate variable tube lengths such that the drift length could be extended or shortened. High voltage was applied on the first electrode of the desolvation tube with a high-voltage DC–DC converter delivering up to 40 kV (40A24-P15-25PPM-F-M-C, UltraVolt, Ronkonkoma, NY).

A three-grid ion shutter, as first reported by Langejuergen,^30^ was placed between the desolvation and drift sections and was composed of etched stainless-steel grids of 0.1 mm thickness separated by 300 μm poly(tetrafluoroethylene) (PTFE) spacers. The grids were obtained from Newcut (Newark, NY) and ordered to the exact same design as detailed by Reinecke and Clowers.^20^ The two outer grids were each tied to a 4700 pF bypass capacitor (Vishay, Farnell, Leeds, UK). These were wired to the middle grid through 150 kΩ resistors (Vishay, Digi-Key Electronics). The injection pulses were created with two FET pulsers, which were obtained from GAA Custom Electronics (www.gaacustom.com). These are battery operated, floated to the high voltage at the gate, and triggered via an optical fiber to maintain the isolation. The circuitries are given in Garcia *et al.*(31) Two of these were wired in series, and the connection point was attached to the middle grid, as described by Butalewicz *et al.*(32) This allowed the control of the electric field at the gate in a two-state or tristate fashion. Triggering was achieved with an Analog Discovery 2 unit (Digilent, Pullman, WA).

At the end of the drift tube, an aperture grid identical to the shutter grids was positioned in front of a Faraday plate detector, separated by a 300 μm spacer. The aperture grid was tied to ground through a 510 kΩ resistor and a 0.22 μF ballast capacitor (Wima, Mouser Electronics, Mansfield, TX). The Faraday plate detector was adopted from the design by Reinecke and Clowers^20^ and was backed by a stainless-steel gas chamber. The latter featured a drift gas inlet through which N_2_ 5.0 (Pangas, Pratteln, Switzerland) was introduced. The drift gas was dried by a molecular sieves filter (SPure H_2_O Filter, BGB Analytik AG, Böckten, Switzerland), and its flow rate was regulated to 500 mL·min^–1^ with a mass flow controller (Bronkhorst, Aesch, Switzerland). The current detected at the Faraday plate was transmitted to the detector circuitry with the help of a short coaxial connector. This circuitry provided a two-stage amplification and consisted of a LMC6001 in the transimpedance amplifier (TIA) configuration followed by a OPA227P (both from Texas Instruments, Digi-Key Electronics). The gains were set to 10^8^ (4.7 × 10^8^ for some amplifier tests) and 10, respectively, leading to a total gain of 10^9^ V·A^–1^. The feedback resistors of 100 and 470 MΩ for the TIA were products of TE Connectivity (RGP0207CHJ100M and RGP0207CHJ470M) obtained from Farnell. The spectra were acquired with a Picoscope 5443D PC oscilloscope (Pico Technology, St. Neots, UK) and filtered with a digital low-pass filter matched to the amplifier bandwidth. The amplifier tests were carried out with the same oscilloscope. The atmospheric pressure in Basel was obtained from the Federal Office of Meteorology and Climatology website (www.meteoswiss.admin.ch), and the temperature in the laboratory was regulated by air conditioning. The voltages were measured using a high-voltage probe (HVP40, Testec, Farnell) connected to a digital multimeter (Fluke, Everett, WA) and the values were corrected for the 1 GΩ input impedance when needed.

Sheathless ESI was implemented using a microfluidic tee as described by Chantipmanee and Hauser^24^ with minor modifications. It is also illustrated in Figure 1. The emitter consisted of a 60 mm long fused silica capillary of 50 μm internal diameter (TSP-050375-M-10, BGB Analytik AG). The standard solutions were pumped with a syringe pump (KDS 100 legacy, KD Scientific, Holliston, MA) at a flow rate of 50 μL·h^–1^ through a 35 cm long PTFE tubing to the tee. Disposable syringes with Luer-lock were enclosed in a polypropylene sleeve for additional electrical isolation. High voltage from a DC–DC converter (40A12-P4-E, UltraVolt) was applied to the solution using a stainless-steel pin with 1.6 mm diameter. The assembly was placed on the custom-made PEEK holder featuring a tapered tip supporting the capillary. This electrospray probe was positioned such that the capillary outlet was centered and at a distance of 2 mm from the entrance of the desolvation tube.

### Calculations

The mobility *K* (expressed as cm·V^–1^·s^–1^) was calculated using eq 3, where *t*_d_ is the drift time, *L* is the drift length, and *V*_D_ is the drift voltage
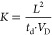
3

The mobility can be normalized with respect to the experimental temperature *T* and pressure *p* yielding the reduced mobility *K*_0_ under standard conditions *T*_0_ and *p*_0_, respectively, of 273.15 K and 1 atm (eq 4)
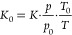
4

The *ideality*, expressed in percent, was calculated as the ratio between the experimentally determined resolving power, *R*_p_, obtained after optimization of the drift voltage and the theoretical value, *R*_opt_, calculated from eq 5(28)
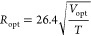
5

### Safety

The voltages used in this work are hazardous, and appropriate measures to prevent accidental exposure of the operator must be taken.

## Results and Discussion

### Effect of the Drift Tube Length and Voltage on the Resolving Power

**Figure 2:**
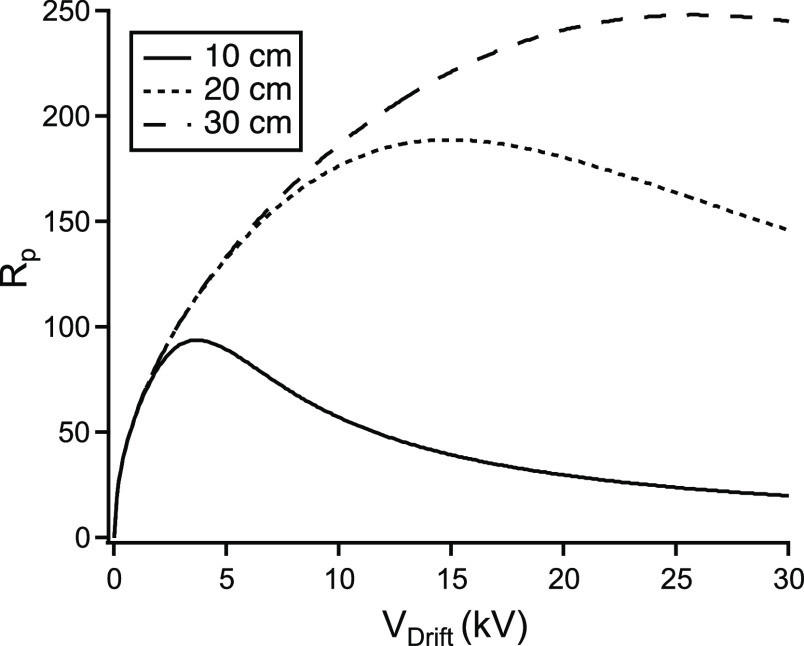
Predicted resolving powers, *R*_p_, according to eq 2, in dependence of the drift tube voltage for three different lengths.

The drift tube length and voltage affect the drift time, and this has a bearing on the separation as well as on the diffusional band broadening represented by the second term in the denominator of eq 2. It is not immediately obvious how these parameters combine to affect the resolving power. However, as illustrated in Figure 2, plotting the theoretically expected resolving power according to eq 2 in dependence of the applied voltage for different drift tube lengths reveals the pattern. Longer drift tubes can be expected to give better resolving powers, but a different optimum in voltage is predicted for each length of drift tube. Note that this is not just a correction to maintain the field strength, but in fact also increasingly higher field strengths are required to reach the optimum, *i.e.*, about 400, 650, and 900 V·cm^–1^ for drift tube lengths of 10, 20, and 30 cm, respectively (the optimum voltage also shows some dependence on the mobility of the analyte ion). From this, it follows that for best resolving power, the length of the drift tube should be extended as much as is compatible with the handling of the increasingly higher required voltage. For designs of IMS instruments reported in the literature, drift tube voltages are typically in the range from 5 to 20 kV. In our work on capillary electrophoresis, using up to 30 kV, we found that working with higher voltages was possible as long as adequate isolation distances are maintained. High voltage modules up to 40 kV are readily available. Note that air is the poorest insulator of all materials present in the system; however, even the relatively high field strengths of up to about 900 V·cm^–1^ employed in the drift tube are well below the threshold for a corona discharge (∼3 kV·mm^–1^)^33^ and electrical arcing, *e.g.*, between the drift electrodes, was therefore not expected and never observed.

**Figure 3:**
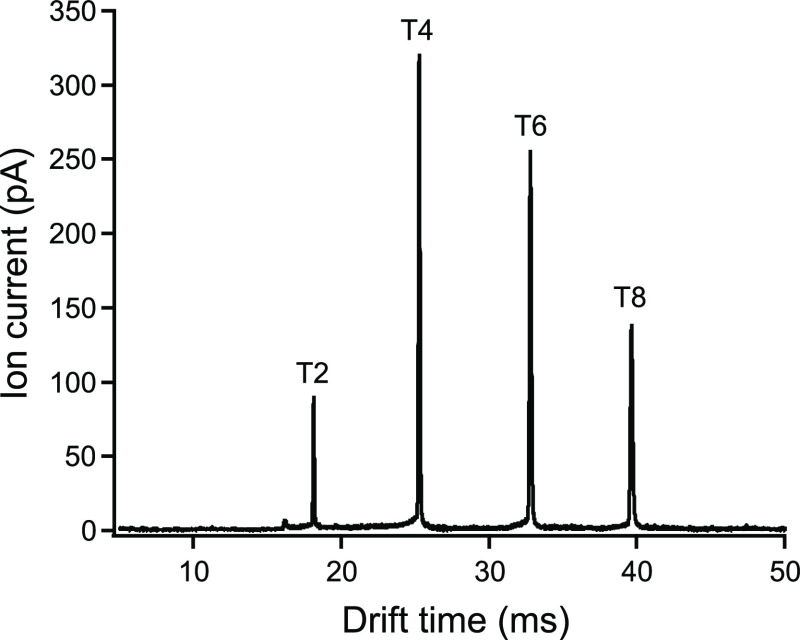
Ion mobility spectra of T2, T4, T6, and T8 at 5 μM for a 100 μs two-state injection. Operating conditions are given in Table 1.

In Figure 3 the separation of four tetraalkylammonium ions used as model compounds^34,35^ with a drift tube length of 30.4 cm and a drift tube voltage of 26.9 kV corresponding to the theoretical optimum according to eq 2 is shown. Further experimental parameters are given in Table 1. The resolving powers were calculated according to eq 3. Values for T2, T4, T6, and T8 ranged from 196 to 228 and are summarized in Table 3. Reduced mobilities were in good agreement with the literature values.^24,36^

**Table 1:** Default Operating Parameters of the ESI–DTIMS Instrument

ESI–IMS parameters	
Electrospray	
sample flow rate (μL·h^–1^)	50
ESI voltage (kV)	40.0
IMS	
desolvation length (cm)	10.0
desolvation voltage (kV)	35.9
drift length (cm)	30.4
drift voltage (kV)	26.9
drift gas flow rate (mL·min^–1^)	500
temperature (K)	293
pressure (hPa)	982
Two-State Injection	
gate blocking voltage (V)	–150
injection time (μs)	100
Tristate Injection	
gate pushing voltage (V)	+150
pushing time (μs)	100
Ion Detection	
TIA gain (V·A^–1^)	10^9^
TIA bandwidth (kHz)	35
number of scans averaged	200
repetition rate (Hz)	10

In order to achieve this performance in terms of resolving power, careful attention had to be paid also to several other critical design and operating parameters.

### Effect of the Injection Time on the Resolving Power

The injection time can be expected to have a direct bearing on the resolving power, and indeed, it is a parameter in eq 2. Short injection times are, in principle, preferred but also result in a loss of sensitivity. In our case, using ESI, a significant reduction in the signal-to-noise ratio was generally observed if the injection time was reduced to less than 100 μs. For a discussion of this aspect, see Kirk and Zimmermann.^37^

### Effect of the Detector on the Resolving Power

**Figure 4:**
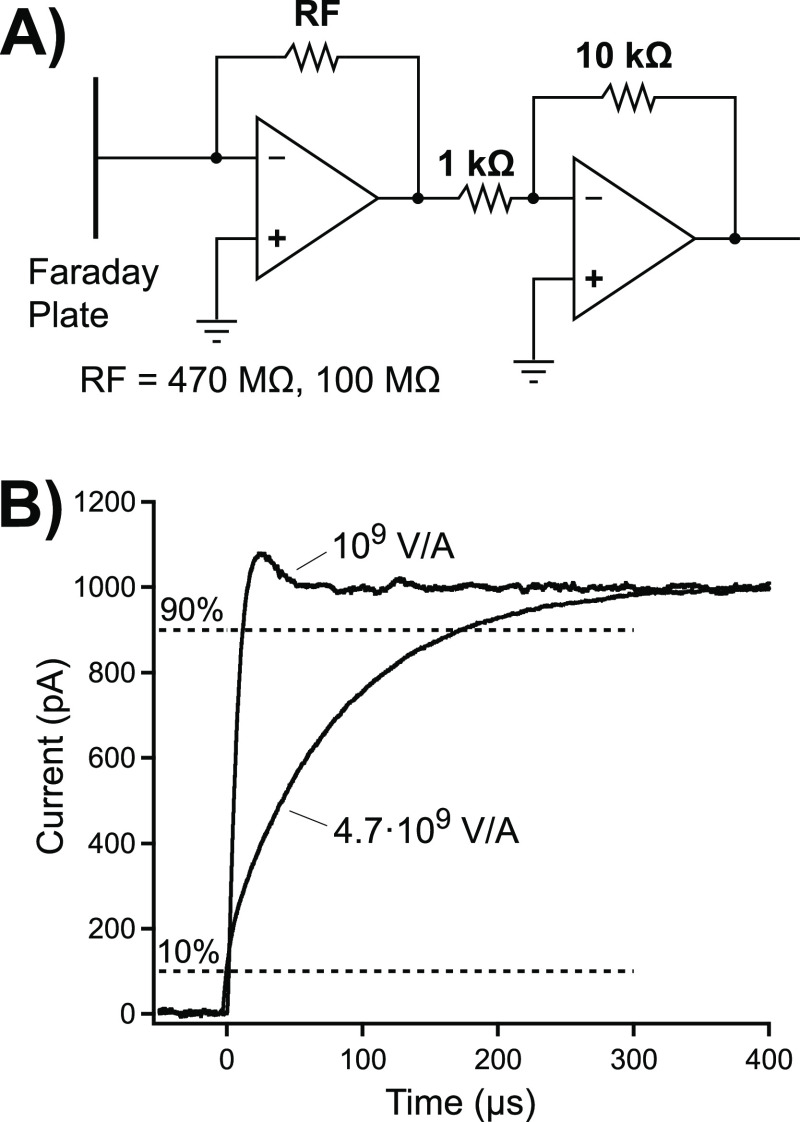
(A) Detector circuitry. (B) Response of the amplifier to a voltage step for 10^9^ and 4.7 × 10^9^ V·A^–1^ gain. The rise time can be read between the 10 and 90% rulers.

Detection in DTIMS is highly challenging as fast transient currents in the picoampere range have to be measured. This requires a transimpedance operational amplifier to convert the current to voltage, which has a low input bias current and low noise, as well as a fairly high bandwidth. As the amplifier employed previously (OPA129 from Texas Instruments) is now obsolete, an alternative was required. Only a few operational amplifiers are available with the required specifications. In preliminary experiments, three candidates were tested, namely, the LMP7721 (Texas Instruments), the ADA4530 (Analog Devices), and the LMC6001 (Texas Instruments). They showed a comparable performance, and the LMC6001 was adopted mainly because it is available in a through-hole package. The circuitry, which includes a secondary voltage amplification stage (×10) to bring the voltage to the desired range for signal acquisition, is shown in Figure 4A. An inadequate response time of the detector circuitry may indeed be adding to the peak width in DTIMS and is therefore also included in eq 2 predicting the resolving power.^27^ The circuitry was tested by applying a square pulse to the amplifier input through a 100 MΩ resistor and recording the amplified signal with an oscilloscope. The response obtained for two different feedback resistors (100 and 470 MΩ, total gains of 10^9^ and 4.7 × 10^9^ V·A^–1^, respectively) is shown in Figure 4B. Note that the response is shown in terms of current, not the raw voltage as measured. As can be seen, the value of the feedback resistor has a strong effect on the response of the detector circuitry. The rise times, *t*_r_, were determined as the time between 10 and 90% thresholds on the rising edge of the signal. The corresponding bandwidths, BW, can be approximated from these rise times as BW = 0.35/*t*_r_.^38^ From the amplifier responses shown in Figure 4B, the rise time and bandwidth were determined to be, respectively, 10 μs and 35 kHz for the 10^9^ V·A^–1^ amplification and 175 μs and 2 kHz for the 4.7 × 10^9^ V·A^–1^ amplification. The speed of the amplifier affects the spectra. For T2, T4, T6, and T8 peak widths (*w*_0.5_) of 93, 114, 146, and 174 μs were obtained for the four ions, respectively, when using the 100 MΩ feedback resistor. As expected, a strong broadening can be observed when using the 4.7 × 10^9^ V·A^–1^ gain. For the higher gain, the respective values were 199, 206, 228, and 250 μs, illustrating the importance of paying close attention to the bandwidth of the detector amplifier. A seemingly small change in the feedback resistance can have a profound effect on the measured signal.

With the lower gain, the width of the narrowest peak, namely T2, was 93 μs and was thus narrower than the injection time of 100 μs. Assuming an insignificant amplifier broadening for the faster amplifier configuration, one can calculate the effectively injected ion cloud width to be 72 μs after subtraction of the diffusional broadening calculated according to eq 3. The injection time difference of 28 μs can be explained by a cumulated effect of the elimination region, *i.e.*, the cutting width is equal to the distance between two grids, and a depletion zone in front of the first grid.^39^ This effect was also observed by Reinecke *et al.* using a similar three-grid shutter.^36^ In the case of the 2 kHz bandwidth amplifier, the FWHM of the T2 peak was measured to be 199 μs. The broadening caused by the slower amplifier can be calculated by solving the denominator of eq 3 for *w*_amp_ and inserting the effective injection width and the diffusion term, leading to a *w*_amp_ of 176 μs. This relates well to the rise time of 175 μs of this amplifier and is thus in good agreement with Kirk who found that *w*_amp_ can be estimated from the rise time, *t*_r_, by multiplication of the latter with a factor of 0.9.

The higher gain setting also caused a reduction in peak height in terms of current. As expected, the impact on the height was more pronounced for the narrower of the four peaks as their edges are steeper and therefore contain higher frequency components than broader ones. Thus, the bandwidth limit of the detector not only affects the resolving power but also has a complicated effect on the signal-to-noise ratio of the measurement.

### Homogeneity of the Field and Geometry of the Drift Tube

The drift tube was created from copper tracks on a polyimide substrate, which was rolled into a tube made from PMMA. The electrode dimensions and their spacing (1.6 mm wide tracks separated by 2.0 mm gaps) were optimized earlier by our group for the rolled-up electrode arrangement by systematic experimental testing of a range of geometries and modeling of the field homogeneity with Simion (Scientific Instrument Services)^24^ and was therefore not investigated again. The geometry arrived at was also found to correspond to dimensions which are typical for stacked drift tubes.^20^ However, please note that, as recently also discussed by Naylor *et al.*,^25^ this aspect is indeed critical for achieving best resolving powers.

The use of a supporting tube, as opposed to just rolling up the polyimide material on its own, was previously reported to improve the resolving power as it improves the rigidity of the setup.^24^ However, it was also found that by inserting the electrode sheet into a tube, rather than rolling it on the outside as in our previous setup, an improvement in resolving power can be achieved. The natural tension of the polyimide keeps the rolled-up material attached to the inner wall of the supporting tube. Presumably, the new arrangement leads to a more uniform electric field in the drift tube as the dielectric constant inside the tube is now homogeneous.

The use of rolled-up electrodes rather than the use of stacked electrodes imparts a possible susceptibility to distortion of the electric field originating from disturbances outside the tube. This arises due to a reduction in shielding because of the lack of depth of the electrode rings.^40^ For this reason, Bohnhorst *et al.*(21) implemented shielding using a staggered two-layer electrode approach in their drift tube based on rigid PCBs, and Smith *et al.*(23) used a dog-leg electrode arrangement for their rolled-up drift tube. For the sake of simplicity, the instrument reported herein was built without such shielding. In the development of the DTIMS device reported herein, its susceptibility was therefore experimentally evaluated by placing a grounded strip of an aluminum sheet (1.4 × 10 × 60 mm) along the 10 cm long drift tube at various distances. A noticeable effect on the resolving power was only observed if the strip was brought to a distance of less than 5 mm from the outside of the drift tube. The resolving powers for T2, T4, T6, and T8 were found to drop from 85 to 70, from 94 to 82, from 91 to 82, and from 87 to 82, respectively, when placing the metal strip directly onto the outer surface of the drift tube. This was accompanied by a similar effect on the peak currents. The effect was therefore only slight, presumably due to the fact that the electrodes are positioned inside a sleeve and that the diameter of 20 mm of the Faraday detector electrode does not cover the entire internal channel width (30 mm). Although no sparking was observed under the test conditions, deliberate placing of a metal object close to the drift tube at high voltage is not recommended in any case, and therefore, it was concluded that electrical shielding is not necessary for the current setup.

A further critical point is the parallelism of injector and aperture grids and of the Faraday detector plates as discussed by Spangler.^29^ Simple mobility calculations reveal that, for example, tilting the collector by 0.5 mm with respect to the drift tube axis would lead to a difference of 70 μs in arrival time for T8 for the present experimental conditions (Table 1). This would greatly impair the performance in terms of resolving power as well as ion losses. Since this level of precision is hard to achieve by visual inspection and manual alignment of the different parts of the cell, the structural components were redesigned in order to ensure geometrical integrity along the drift path. The flanges holding the grids and detector as well as the desolvation and drift tubes were precisely machined at the university machine shop. PEEK was chosen for its stiffness, its chemical resistance, and its dielectric strength. The flanges are now aligned precisely with the help of four rods with accurately machined lengths connecting their corners. The importance of the overall alignment of the DTIMS was investigated using the 10.0 cm separation tube and experimental conditions of Table 2. The detector arrangement, including the aperture grid, was placed in a slight angle such that the Faraday detector plate was tilted by 0.4 mm. As a result, the peak width of T8 was found to be increased by 49 μs, reducing the resolving power from 87 to 76.

**Table 2:** Experimental Conditions for the Determination of the *Ideality* Factor

operating parameters	30 cm drift tube	20 cm drift tube	10 cm drift tube
ESI voltage (kV)	40.0	24.1	12.5
desolvation length (cm)	10.0	10.0	10.0
desolvation voltage (kV)	35.9	20.0	8.4
drift length (cm)	30.4	20.2	10.0
drift voltage (kV)	26.9	13.3	4.2
gate blocking voltage (V)	–150	–115	–75
injection time (μs)	100	100	200
gate pushing voltage (V)	+150	+150	+150
pushing time (μs)	100	100	200

### Electrospray

To create a stable spray, the electrospray voltage was held at a potential of 4.1 kV above the desolvation tube inlet voltage. The ESI potential was, therefore, at 40 kV with respect to ground. This was the highest possible voltage with the DC–DC converter used. As the sample solution is propelled with a syringe pump, special measures had to be taken to avoid any spurious path to electrical ground through the syringe. This may otherwise cause electrophoretic migration of ions in the transfer tube as well as Faradaic reactions leading to unwanted gas evolution. First of all, a metal-free syringe was employed, and in addition, this was contained in a polymeric sleeve for further insulation. The length of the tubing connecting the syringe to the electrospray head was also extended. Without these precautions, the ion current on the IMS detector was found to strongly fluctuate, probably due to an unstable sample flow rate and voltage at the ESI emitter. In addition, the design of the electrospray head was critical due to the high voltage used. A plastic conical tip was used to guide the flexible capillary, which otherwise had a tendency to vibrate on application of the high voltage (Table 3).

**Table 3:** Drift Times, Reduced Mobilities, and Resolving Powers of the Measured Compounds (Average Values for *n* = 5; the Uncertainties Are Standard Deviations)

compound	drift time *t*_d_ (ms)	reduced mobility *K*_0_ (cm^2^ V^–1^ s^–1^)	resolving power *R*_p_
T2	18.15 ± 0.07	1.71	195.7 ± 6.0
T4	25.30 ± 0.08	1.23	221.0 ± 3.1
T6	32.84 ± 0.10	0.95	224.8 ± 3.1
T8	39.68 ± 0.11	0.78	227.9 ± 4.2
T10	45.73 ± 0.01	0.69	214.1 ± 1.3
T12	49.74 ± 0.01	0.63	227.0 ± 1.4
BAC-C12	29.82 ± 0.01	1.06	210.5 ± 0.4
BAC-C14	31.22 ± 0.01	1.01	213.4 ± 1.6
BAC-C16	32.37 ± 0.01	0.98	217.1 ± 2.4

### Ideality

**Figure 5:**
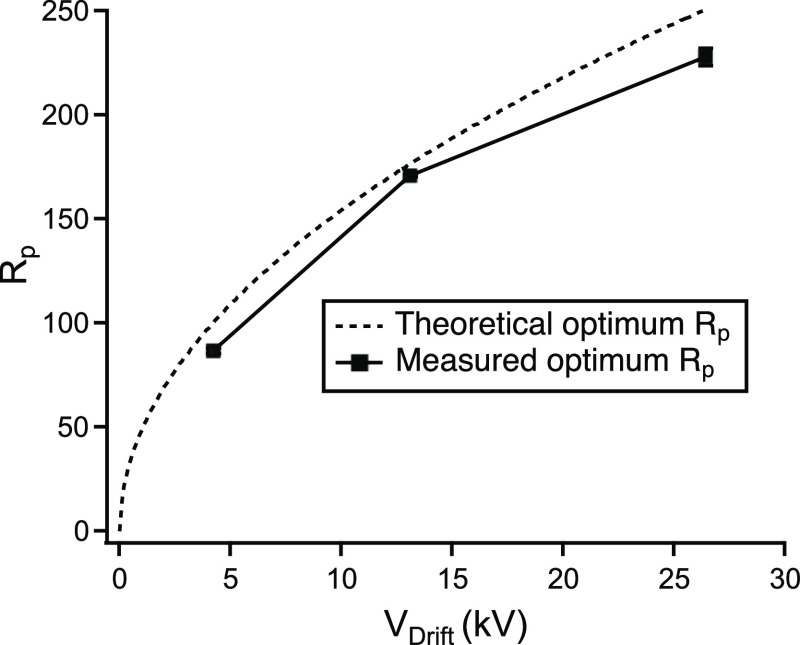
Measured optimum resolving powers, *R*_p_, obtained for the three experimental conditions listed in Table 2 compared to the theoretical values according to eq 4.

The measured resolving powers (for T8 at 5 μM) for the optimum voltage obtained for the 30.4 cm drift tube as well as for shorter drift tube lengths of 20.2 and 10.0 cm are shown in Figure 5, together with a plot of the theoretically achievable value according to eq 5 (the latter correspond to the maxima in Figure 2). It can be seen that the measured values (228, 172, and 93, respectively) closely follow the curve of the theoretical optimum resolving power. Zimmermann and co-workers introduced the *ideality*, the ratio between the two values, as a parameter for evaluating the performance of a DTIMS instrument in terms of resolving power.^28^ The *ideality* factors corresponding to the three measurements are 86, 91, and 97%.

Note that Smith *et al.*(23) achieved an *ideality* of 95.6% for their flexible drift tube, but Naylor *et al.*(25) reported an *ideality* of only 70% for a flexible drift tube. However, when using a stacked PCB IMS with comparable electrode pitch and width as in the present work, Naylor *et al.* also determined an *ideality* of 90%. These results suggest again, first, that the electrode dimensions and distances are critical in achieving high resolving powers, and second, that the use of the rolled-up drift tube with an optimized electrode geometry does not incur a penalty in terms of resolving power.

### Ion Gating: Two State *vs* Tristate

The use of tristate injection has been suggested in order to minimize a discrimination of ions during the injection. The working principle is described elsewere.^32,41,42^ To investigate the possibility of tristate injection on the new instrument, the pulsing regime was implemented by combining two pulser circuitries as reported by the Clowers’ group.^32^ Peak ion currents and resolving powers obtained for the lower-mobility ions, namely, T8, T10, and T12, were compared and are tabulated in Table 4. These compounds were introduced separately to avoid any possible bias in the measurements by ion suppression effects affecting ESI. With tristate injection, the gate depletion zone was reduced compared to the two-state scheme, and more ions were injected. Consequently, the detected ion current and the peak widths were increased. A shortening of the injection pulse was, therefore, required to recover the separation performances. By cutting back the tristate injection time to half of the two-state injection time, the resolving powers could be maintained for T8 and T12 and was less than 5% lower for T10. Although it is likely that the resolving power was still limited by the injected width, the injection time was not further shortened as it led to weak signals, from which the resolving power could be poorly extracted. Thus, resolving powers of 222.8 ± 2.5 and 227.0 ± 1.4 were obtained for T10 and T12, respectively, using the tristate injection.

**Table 4:** Ion Currents and Resolving Powers of T8, T10, and T12 for Two-State and Tristate Injections (Average Values for *n* = 5; the Uncertainties Are Standard Deviations)

		two-state		tristate	
compound	injection time (μs)	ion current (pA)	resolving power *R*_p_	ion current (pA)	resolving power *R*_p_
T8	100	134.5 ± 5.1	227.9 ± 4.2	248.8 ± 11.8	211.6 ± 1.1
	50			148.2 ± 26.7	228.7 ± 1.0
T10	100	92.3 ± 1.1	230.9 ± 5.0	223.1 ± 4.6	214.1 ± 1.3
	50			111.7 ± 3.6	222.8 ± 2.5
T12	200	98.2 ± 8.3	225.8 ± 3.9	136.9 ± 16.1	182.2 ± 2.6
	100			101.0 ± 11.8	227.0 ± 1.4

### Validation

**Figure 6:**
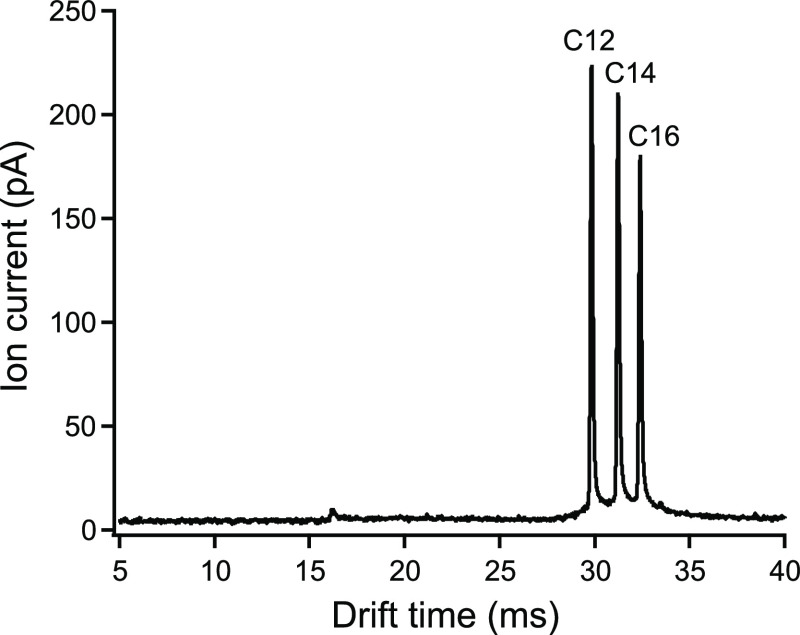
Ion mobility spectrum of a mixture of BAC-C12, BAC-C14, and BAC-C16 at 5 μM for a 100 μs two-state injection. Conditions are given in Table 1.

The performance of the instruments was further tested with three benzalkonium salts, which differ only slightly in their structure and size and therefore present a challenge to separation. A standard mixture of BAC-C12, BAC-C14, and BAC-C16 was separated. The spectrum for a 100 μs two-state injection is depicted in Figure 6. In this case, the resolving powers were found to be all higher than 210, confirming the improvement of the IMS instrument with respect to this parameter. The values are included in Table 3 together with the reduced mobilities. From the spectrum obtained for the mixture at 5 μM each, it can be estimated that the limit of detection for these substances at the operating conditions employed is below 1 μM.

## Conclusions

Careful optimization of a drift tube of an ESI–DTIMS instrument yielded resolving powers of up to 228 for singly charged ions. This, to our knowledge, is the highest reported value achieved with ESI for *z* = 1. The *ideality* factor of the drift tube was better than 86%, independent of its length. This was achieved with a drift tube based on rolled-up electrodes and demonstrates that this facile construction approach can lead to instruments with state-of-the-art resolving powers. The modestly short injection times between 50 and 200 μs required are not challenging the shutter electronics. Despite the extended drift distance, the IMS cell has a reasonable total length of approximately 60 cm with the ESI probe. Moreover, even if the improved DTIMS was operated at relatively high voltages, electric insulation of the device was still manageable.
